# Subnanomolar Detection of Oligonucleotides through Templated Fluorogenic Reaction in Hydrogels: Controlling Diffusion to Improve Sensitivity

**DOI:** 10.1002/anie.201701356

**Published:** 2017-04-06

**Authors:** Dana Al Sulaiman, Jason Y. H. Chang, Sylvain Ladame

**Affiliations:** ^1^ Department of Bioengineering Imperial College London South Kensington Campus London SW7 2AZ UK

**Keywords:** fluorescence, hydrogels, nucleic acids, oligonucleotide-templated reactions, PEBBLE sensors

## Abstract

Oligonucleotide‐templated reactions are valuable tools for nucleic acid sensing both in vitro and in vivo. They are typically carried out under conditions that make any reaction in the absence of template highly unfavorable (most commonly by using a low concentration of reactants), which has a negative impact on the detection sensitivity. Herein, we report a novel platform for fluorogenic oligonucleotide‐templated reactions between peptide nucleic acid probes embedded within permeable agarose and alginate hydrogels. We demonstrate that under conditions of restricted mobility (that is, limited diffusion), non‐specific interactions between probes are prevented, thus leading to lower background signals. When applied to nucleic acid sensing, this accounts for a significant increase in sensitivity (that is, lower limit of detection). Optical nucleic acid sensors based on fluorogenic peptide nucleic acid probes embedded in permeable, physically crosslinked, alginate beads were also engineered and proved capable of detecting DNA concentrations as low as 100 pm.

Widely present in nature, most notably to catalyze phosphodiester bond formation in key biological processes including transcription and translation, oligonucleotide templated reactions (OTRs) are highly versatile and have been successfully applied to a broad range of chemistries.^1^ This powerful concept allows unfavorable reactions between molecules present at very low concentrations to take place by increasing their effective concentration. OTRs rely upon sequence‐specific Watson–Crick base‐pairing to bring together two reactive moieties (or probe‐heads), each attached to the end of an oligonucleotide (or oligonucleotide analogue) strand (Scheme 1).^1^ Because of their intrinsic specificity and high programmability, OTRs have found valuable applications in controlled organic synthesis,^2^ DNA‐encoded chemistry for drug discovery,^3^ and nucleic acid (NA) sensing both in vitro1b,1e,1f, 4 and in vivo.^5^ For sensing applications, OTRs were successfully engineered whereby only the NA target of interest acts as a template to catalyze an otherwise highly unfavorable reaction, which can be monitored optically (for example, changes in fluorescence intensity and/or wavelength). In such cases, the fluorescence intensity emitted by the product of the OTR is directly proportional to the amount of NA target present, whilst only very low levels of background fluorescence can be detected in the absence of the template (leading to a high signal‐to‐noise ratio or S/N). Representative examples include the quenched auto‐ligation (QUAL)4b, 5a,5b strategy in which an OTR causes the release of a quencher molecule, resulting in the restoration of the intrinsic fluorescence of an otherwise quenched nearby fluorophore. Another common strategy uses an oligonucleotide template to catalyze the formation of a fluorescent dye from two non‐ or weakly fluorescent precursors.4c, 6 Some of the reactions most commonly used in such applications include ester hydrolyses,^7^ nucleophilic substitutions,4a, 8 aldolizations,^6^ tetrazine ligations,5j,5k and the Staudinger reaction.5e,5i

**Scheme 1:**
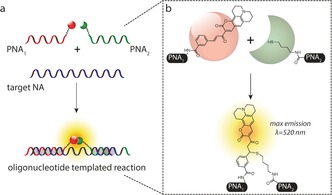
a) Sensing strategy based on the concept of oligonucleotide‐templated reaction (OTR). Watson–Crick base‐pairing between a target NA and two complementary engineered PNA probes catalyzes a fluorogenic reaction between two probe heads on the N‐terminus of PNA_1_ (PNA‐coumarin) and the C‐terminus of PNA_2_ (PNA‐thiol), respectively. b) Fluorogenic Michael addition between an aliphatic thiol and a quenched coumarin. The unquenched coumarin has a maximum emission at *λ*=520 nm.

For optimal sensitivity and specificity, peptide nucleic acid (PNA)‐based probes have been commonly used as an alternative to standard oligonucleotides. They offer the advantages of having a higher affinity for complementary DNA (or RNA) and being more responsive to single nucleotide mutations.^9^ To further improve sensitivity and achieve lower limits of detection (LOD), it is often required to introduce additional amplification steps, which typically involve either enzymatic target amplification using polymerases as in PCR^10^ and/or signal amplification (that is, catalytic turnover in which the same NA template is capable of catalyzing more than one chemical reaction).5j, 11 Sensitivity can however be compromised by background noise originating from either incomplete quenching of the fluorogenic probe heads before addition of template or non‐templated reactions between probes occurring even in the absence of NA target. Herein, we demonstrate the novel use of OTR within permeable hydrogels, whereby the sensitivity and specificity can be enhanced through the control of diffusion. Probes encapsulated by biologically localized embedding (PEBBLE) sensors were also engineered and validated in vitro. We have previously reported the development and validation of fluorogenic PNA probes for the amplification‐free sensing of cancer‐specific circulating microRNA (miR) biomarkers from patients’ blood (Supporting Information, S1).^12^ Briefly, miR sensing relied on a fluorogenic RNA‐templated Michael addition reaction between an aliphatic thiol and a quenched coumarin immobilized at the C‐terminus and the N‐terminus of PNA probes, respectively (Scheme 1). Herein, we explored the possibility to carry out fluorogenic OTR within media of varying physical properties that can affect probes’ mobility, particularly in hydrogels. Hydrogels are three‐dimensional macromolecular networks of polymer chains that can absorb up to thousands of times their weight in water.^13^ Owing to their water‐imbibing and tailorable properties, they have been used in many biomedical and pharmaceutical applications (for example, tissue engineering).^14^ In this study, agarose and alginate hydrogels were chosen as model systems, being both low‐cost and easily crosslinked under mild conditions that are compatible with OTRs. Both hydrogels formed under near physiological salt concentration and pH, without the need for potentially toxic and DNA‐damaging crosslinking agents.^13^, 14b Both algae‐extracted polysaccharides are physically crosslinked hydrogels such that polymer chains are held by intermolecular forces (Scheme 2).

**Scheme 2:**
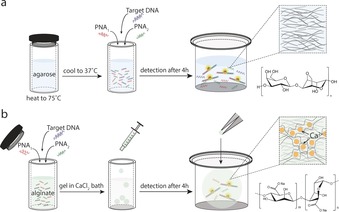
Schematic of the experimental setup for OTR in either a) agarose or b) alginate hydrogels (for experimental details, see Supporting Information, S2). Briefly, a stoichiometric mixture of PNA probes in the presence or absence of NA target was added to a buffered (10 mm potassium phosphate pH 7.4) solution of either agarose or alginate. Gelation was induced almost instantaneously by either rapid cooling (for agarose) or immersion in a calcium chloride bath (for alginate). Reaction efficiency was monitored by fluorescence spectroscopy (*λ*_ex_=485 nm, *λ*_em_=520 nm).

As a proof‐of‐concept, we first investigated the ability to conduct fluorogenic OTR within either agarose (0.33 % w/v) or calcium alginate (1 % w/v alginate, 50 mm Ca^2+^) hydrogels. Control experiments were also performed in aqueous buffered solution and in highly viscous solutions of low percentage agarose (0.1 % w/v) and non‐crosslinked alginate (1 % w/v alginate, no added Ca^2+^). In a typical experiment, both PNA probes (5 μm each) and the target oligonucleotide (1 μm) were added to each medium type and buffered to pH 7.4 with potassium phosphate buffer (10 mm). The two hydrogels were then rapidly gelled, either by rapid cooling to room temperature in the case of agarose or by ionic crosslinking in CaCl_2_ (50 mm) in the case of alginate (Supporting Information, S3). Upon crosslinking, all three reaction components were embedded (with restricted mobility) within the fibrous matrix. After 4 h from reaction initiation, equilibrium was reached in all media (Supporting Information, S4) and the fluorescence intensity was recorded using a Fluorostar fluorescence plate reader (BMG LabTech) (*λ*_ex_=485 nm, *λ*_em_=520 nm). For each condition, the background fluorescence intensities from the PNA‐coumarin probe alone (B) and that from the non‐templated control (NTC) reaction between both PNA probes in the absence of oligonucleotide target were recorded. OTR efficiency was assessed by measuring the ratio between the fluorescence intensities of reactions, at equilibrium, carried out in the presence (OTR) and in the absence (NTC) of oligonucleotide target (that is, the S/N ratio).

Results shown in Figure 1 a demonstrate that OTR can be conducted in media with restricted mobility, including polysaccharides in their non‐crosslinked (that is, highly viscous solution) and crosslinked (set hydrogel) forms. Under these conditions, comparable OTR efficiencies were obtained in agarose and in solution. Reactions performed in alginate resulted in a significant loss of fluorescence intensity, mostly when crosslinked in its hydrogel form. This drop can be partly explained by optical effects (for example, scattering and absorption) affecting the measured fluorescence emitted by both quenched and unquenched coumarin probes in this medium. The contribution of these optical effects on the absolute fluorescence of the unquenched coumarin (product of the OTR, 1 μm target) in different media was examined specifically (Supporting Information, Figure S5). While the fluorescence intensity was very slightly decreased in agarose (up to 1.2‐fold) compared to that in solution, the decrease was much more significant in non‐crosslinked (by ca. 1.6‐fold) and crosslinked (by ca. 6.2‐fold) alginate. After correcting for these optical effects (Figure 1 b), it became apparent that the reaction efficiency in the absence of target (that is, NTC) was significantly reduced in hydrogels (compared to similar reactions carried out in solution). This may be due to the fibrous matrix in hydrogels, which prevents non‐specific interactions between the probes through steric hindrance and restricted diffusion. Although diffusion of small molecules in agarose has already been explored by others,^15^ we briefly investigated how the diffusion of PNA probes was specifically affected in such a medium. For this purpose, a fluorescein‐tagged PNA (MW=2607 Da) with a sequence similar to that of PNA_1_ was synthesized (Supporting Information, S1). Its diffusion coefficient in 0.8 % (w/v) agarose was 1.2×10^−10^ m^2^ s^−1^, which is up to 50 % lower than the diffusion coefficient of similar molecules in solution (Supporting Information, S6).^16^ Although our probes can diffuse in hydrogels, they experience hindered diffusion, which could be at least partly responsible for the reduction in NTC efficiency in either viscous solutions or crosslinked hydrogels. In the case of alginate, additional effects such as electrostatic interactions between the positively charged probes and negatively charged alginate fibers may also contribute towards hindered diffusion.

**Figure 1:**
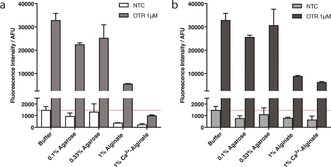
a) Plot of fluorescence intensity (*λ*_ex_=485 nm, *λ*_em_=520 nm) of a stoichiometric mixture of PNA probes (5 μm each) in the absence (NTC) or in the presence (OTR) of 1 μm target DNA, after 4 h incubation in five different media (experiments conducted in triplicate): potassium phosphate buffer (10 mm, pH 7.4); highly viscous agarose solution (0.1 % w/v); set agarose hydrogel (0.33 % w/v); highly viscous alginate solution (1 % w/v); and set alginate hydrogel (1 % w/v crosslinked with 50 mm CaCl_2_). The fluorescence intensity of each buffered medium alone (with no added probe) was also recorded and subtracted from all the above readings. b) Fluorescence intensity of OTR and NTC from Figure 1 a corrected for optical effects (light scattering and absorption) (Supporting Information, S5).

Typically, OTRs are performed using low reactant concentrations (low micromolar range) so that non‐specific reactions in the absence of template are highly unfavorable. When applied to NA sensing, keeping the NTC efficiency low ensures low background noise and high S/N ratio. The effect of increasing the concentration of the probe on NTC efficiency in the various media was therefore investigated. Increasing probe concentrations from 3 to 15 μm resulted in a gradual increase in fluorescence intensity for the NTC in buffer solution, as expected for a bimolecular reaction. However, this effect was greatly attenuated in the agarose hydrogel (for example, by ca. 3.5‐fold at a probe concentration of 15 μm, Figure 2 a). In alginate‐based media, no fluorogenic reaction was detectable in the absence of added template, even at the highest probe concentration tested (Figure 2 a). Accordingly, OTR efficiency (S/N ratio) was found to be negatively affected by increasing probe concentration in buffer solution and, to a lesser extent, in agarose, mainly owing to an increase in background noise. However, the S/N ratio remained mostly unaffected in alginate (Figure 2 b). Building on the observation that NTC efficiency is reduced in hydrogels, we next investigated whether increasing the probe concentration could improve the sensitivity of NA sensing by fluorogenic OTR. Sensing reactions were first assessed using a fixed concentration of probes (3 μm each) and varying the DNA concentration from 1 nm to 1 μm. A linear correlation between fluorescence intensity and DNA concentration was observed in solution and in both hydrogels (agarose 0.33 % (w/v) and crosslinked Ca^2+^‐alginate; Supporting Information, S7). Under those conditions, a similar LOD of circa 200 nm was observed for each of the media tested. Probe concentration was then increased up to 9 μm. This resulted in a higher LOD (that is, decreased sensitivity) in buffer from 200 nm to 1 μm, mainly caused by the increase in background noise. In agarose, a similar (although weaker) effect was observed with the LOD increasing to 700 nm when the probe concentration was increased from 3 to 9 μm. On the contrary, a significant improvement in sensitivity was achieved in alginate media, with the LOD improving to 50 nm in alginate hydrogel (at a probe concentration of 9 μm). In the case of non‐crosslinked alginate (Figure 3), an even greater improvement in sensitivity was achieved, in the subnanomolar range. More specifically, 2‐ and 20‐fold decreases in LOD were observed after doubling and tripling the probe concentration, respectively.

**Figure 2:**
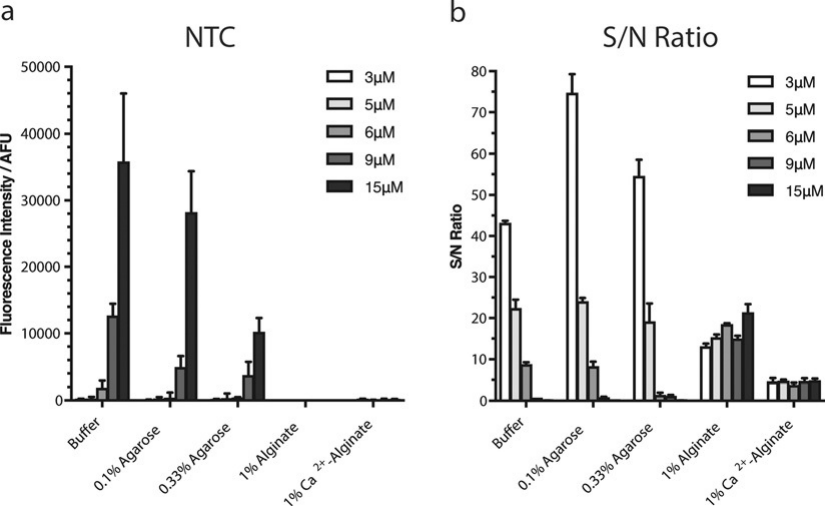
a) The effect of increasing the PNA probe concentration (from 3 to 15 μm) on NTC efficiency in five different media (experiments conducted in triplicate): potassium phosphate buffer (10 mm, pH 7.4); highly viscous agarose solution (0.1 % w/v); set agarose hydrogel (0.33 % w/v); highly viscous alginate solution (1 % w/v); and set alginate hydrogel (1 % w/v crosslinked with 50 mm CaCl_2_). Fluorescence intensities were corrected by the fluorescence intensity of the PNA‐coumarin alone to display only the signal originating from the product of the NTC. b) S/N ratio for OTRs in the presence of 1 μm DNA using increasing concentrations of PNA probes. S/N ratio is defined as the ratio between the fluorescence intensity of the OTR and that of the corresponding NTC.

**Figure 3:**
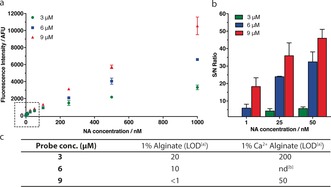
a) Effect of varying probe concentration (3, 6, and 9 μm) and varying DNA target concentration (from 1 nm to 1 μm) on fluorescence intensity in 1 % (w/v) non‐crosslinked alginate (experiments conducted in triplicate); b) S/N ratio of OTRs at probe concentrations of 3, 6, and 9 μm and target NA concentrations of 1, 25, and 50 nm; c) calculated LOD^[a]^ (nm) for OTR‐based NA sensing in alginate media. [a] LOD=3×SD(NTC)+NTC, [b] not determined.

Finally, a PEBBLE NA sensor based on fluorogenic PNA probes embedded within a permeable alginate hydrogel was designed and tested. Briefly, spherical alginate beads (with a diameter of ca. 5 mm) were engineered containing a stoichiometric mixture of PNA probes (10 μm each). The beads were then incubated in solutions containing varying concentrations of DNA target (from 0 to 1 μm). After 4 h incubation, the beads were removed from the solution and washed extensively before being imaged using a fluorescence scanner (Typhoon FLA9500; Figure 4).

**Figure 4:**
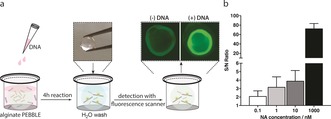
a) Schematic of alginate PEBBLE NA sensors. Beads containing a mixture of PNA probes (10 μm each) crosslinked in a 50 mm CaCl_2_ bath (5 min) were incubated in solutions with target NA. After 4 h, the beads were removed, washed, and imaged with a Typhoon FLA9500 fluorescence scanner (GE Healthcare). Images were analyzed using FIJI/ImageJ. The average hydrogel bead diameter was 5 mm. Fluorescence scanner images are of beads after incubation in solution with (right) or without (left) 1 μm DNA target. b) S/N ratio of alginate PEBBLEs after incubation in solutions containing 0.1, 1, 10, and 1000 nm target DNA (*n*=3).

The beads incubated in a solution containing 1 μm DNA showed a fluorescence intensity up to 80‐fold higher than that of beads incubated in the DNA‐free solution. This result clearly demonstrates that, whilst PNA probes remain trapped within the hydrogel bead (through entanglement and/or electrostatic interactions with the hydrogel fibers), DNA molecules can diffuse from the solution to within the hydrogel, where the fluorogenic OTR then takes place. In terms of sensitivity, this alginate NA sensor proved capable of detecting DNA concentrations as low as 100 pm (Figure 4 b). Comparable LODs (in the range 0.1–10 nm) were previously reported by others for OTRs based on alternative NA‐templated fluorogenic chemistries, with similar reaction times.1e, 11e Lower LODs down to 0.5 pm have been previously reported, but those reports relied on template turnover for signal amplification and required significantly longer reaction times (>7 h).5j, 11b

In summary, the first examples of fluorogenic OTR carried out in physical hydrogels (agarose and alginate) are reported. Of particular interest for sensing applications, performing OTRs in hydrogels significantly reduces background noise originating from non‐templated reaction between the probes in the absence of NA target. This accounts for the higher S/N ratio and lower LOD (subnanomolar) when compared to similar reactions carried out in buffer solution, especially at unusually high probe concentrations. PEBBLE NA sensors based on fluorogenic PNA probes embedded within alginate beads were also tested and proved capable of detecting DNA concentrations as low as 100 pm. While the LOD and reaction times are comparable to existing hybridization‐based optical assays using surface‐bound PNA probes,^17^ these PEBBLE NA sensors offer the advantages of being label‐free and requiring no covalent immobilization of the probes, making them much simpler to implement. Although significantly lower LODs can be achieved by PCR‐based techniques, we recently demonstrated that this enzyme‐free, isothermal technology was sensitive enough to detect endogenous concentrations of circulating miRNAs in human blood, without the need for any amplification step and for a fraction of the cost.^12^ Further improvement of the LOD by embedding the probes into permeable hydrogels will ensure that even the least abundant miRNAs can be detected. Permeable hydrogels such as agarose or alginate could present themselves as low‐cost, easily tunable matrices for the next generation optical NA sensors. Exploration of biomedical applications based on this technology is currently underway in our laboratory.

## Conflict of interest

The authors declare no conflict of interest.

## Supporting information

As a service to our authors and readers, this journal provides supporting information supplied by the authors. Such materials are peer reviewed and may be re‐organized for online delivery, but are not copy‐edited or typeset. Technical support issues arising from supporting information (other than missing files) should be addressed to the authors.

Supplementary
