# Supplementary material for: Subnanomolar Detection of Oligonucleotides through Templated Fluorogenic Reaction in Hydrogels: Controlling Diffusion to Improve Sensitivity
Source: Angew Chem Int Ed Engl. 2017 Apr 6;56(19):5247–51. doi: 10.1002/anie.201701356 (PMC5502887; doi:10.1002/anie.201701356)
Supplement: Supplementary file 1 — Supplementary [file ANIE-56-5247-s001.pdf]

Angewandte Chemie
Eine Zeitschrift der Gesellschaft Deutscher Chemiker

Supporting Information

# Subnanomolar Detection of Oligonucleotides through Templated Fluorogenic Reaction in Hydrogels: Controlling Diffusion to Improve Sensitivity

*Dana Al Sulaiman, Jason Y. H. Chang, and Sylvain Ladame**

anie_201701356_sm_miscellaneous_information.pdf

# Supporting Information

## Table of Contents

**S1. PNA probe structures and characterisation via MALDI-TOF** **2**

*$PNA_1$: PNA-coumarin probe* *2*

*$PNA_2$: PNA-thiol probe* *2*

*PNA-Fluorescein probe* *3*

**S2. OTR in agarose and alginate media** **4**

*Agarose* *4*

*Alginate* *4*

*Fluorescence reading* *4*

**S3. Optimisation of Alginate Hydrogel Conditions** **5**

**S4. Kinetics of OTR in all media** **6**

**S5. Optical Effects on measured fluorescence** **7**

**S6. Estimating diffusion coefficient of PNA probes in Agarose** **8**

**S7. Effect of Increasing probe concentration on LOD in 0.33% Agarose, 1% $Ca^{2+}$-Alginate and 1% Alginate** **9**

**S8. References** **9**

# S1. PNA probe structures and characterisation via MALDI-TOF

HPLC purified DNA oligonucleotide (141-DNA, $^{5'}$AACACTGTCTGGTAAAGATGG$^{3'}$) was purchased from Invitrogen (UK).
PNA probes were synthesised as previously reported by us.[1]

| Probe | Sequence (C-terminal to N-terminal) | MW (Da) |
|---|---|---|
| $PNA_1$ | Arg-Arg-TGTGACA-Coumarin | 2643.69 |
| $PNA_2$ | SH-CATTTCT-Arg-Arg | 2258.99 |
| PNA-fluorescein | Arg-Arg-TGTGACA-fluorescein | 2606.59 |

## $PNA_1$: PNA-coumarin probe

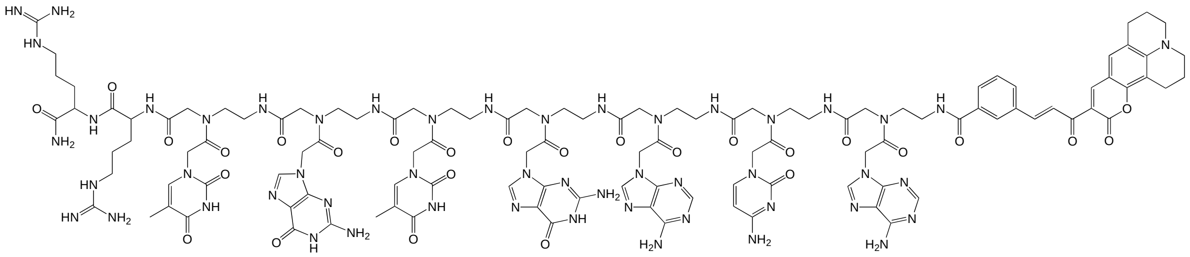

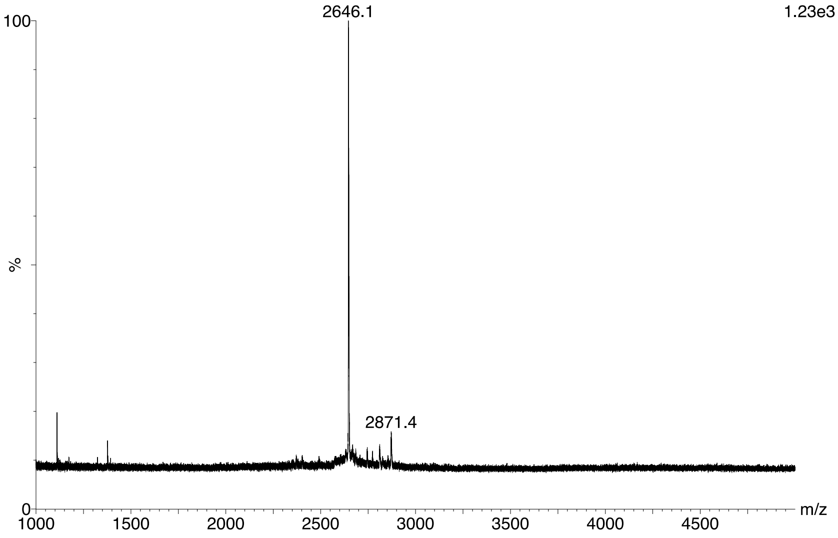


## $PNA_2$: PNA-thiol probe

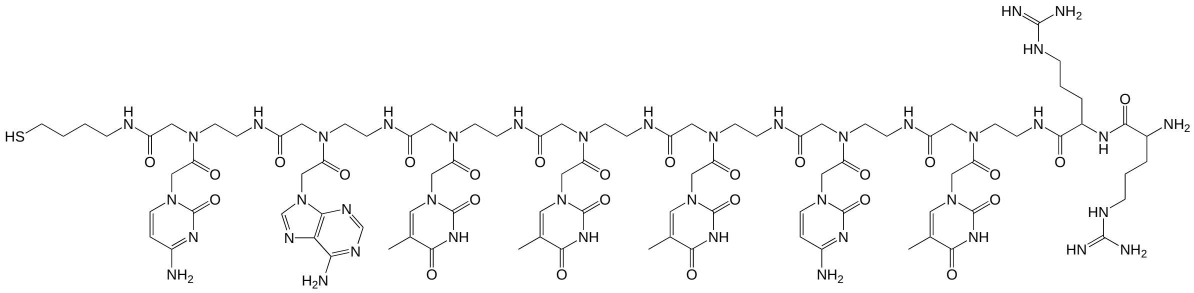

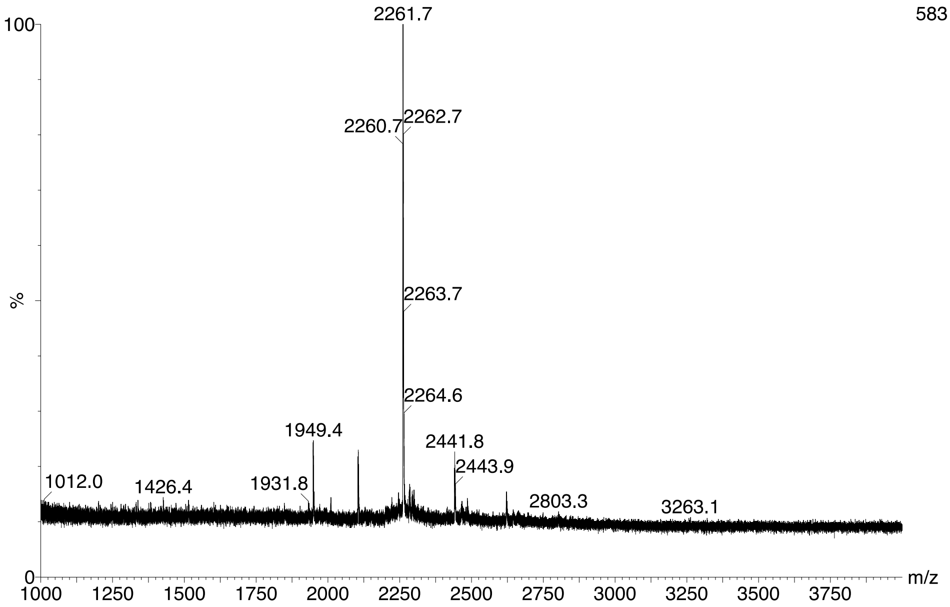


## PNA-Fluorescein probe

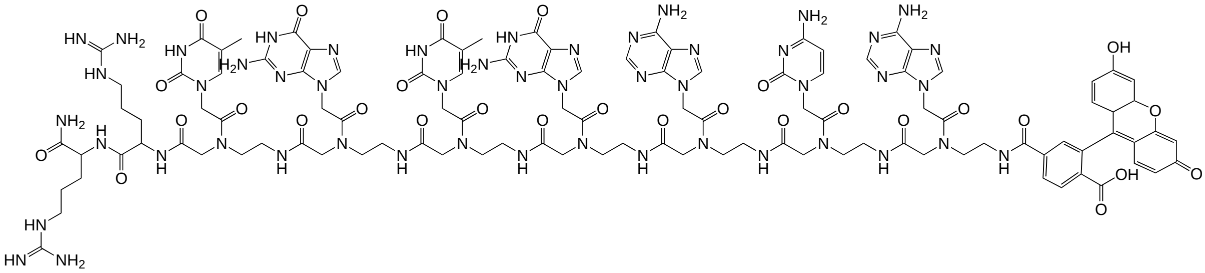

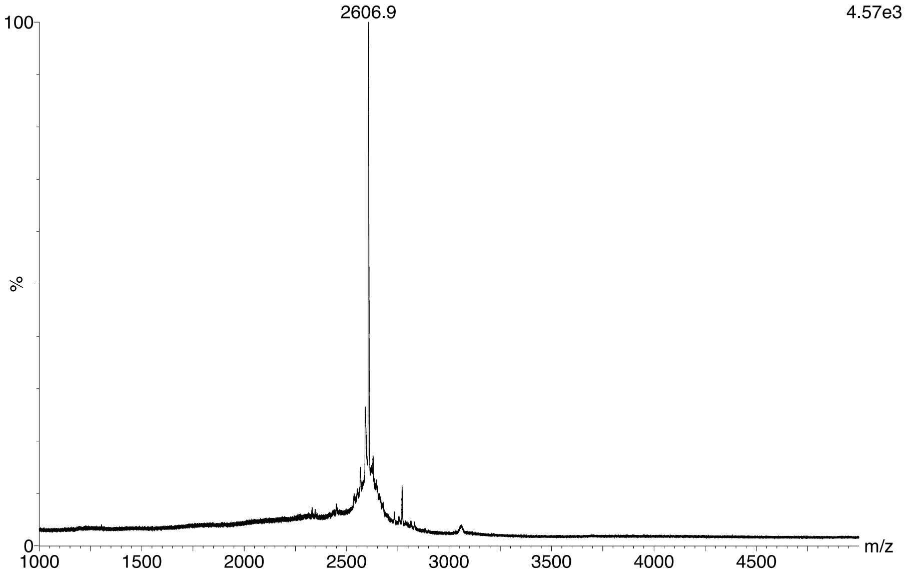

# S2. OTR in agarose and alginate media

## Agarose

Agarose gels were prepared by measuring out an amount of low melting point agarose (Sigma-Aldrich) into an appropriate amount of DNAse- RNAse-free water to make up the desired concentration of 0.1% (w/v) or 0.33% (w/v). Agarose was dissolved by heating up the suspension in a microwave for 40 s then leaving it to cool down to 37ºC (just above gelation temperature). At this temperature, the PNA probes only (for NTC) and probes with target DNA (for OTR) were added and buffered to pH 7.4 with potassium phosphate buffer (10 mM). 90 μl aliquots of this mixture were cast into wells of a 384-well microplate and let to set at rt.

## Alginate

Alginate gels were prepared by measuring out an amount of low-viscosity alginic acid sodium salt from brown algae (Sigma-Aldrich) into an appropriate amount of DNAse- RNAse-free water to make up the desired concentration of 2% (w/v). The alginic acid was dissolved by heating up the suspension in a microwave for 20 s then leaving it to cool down to rt. Appropriate volumes of the PNA probes only (for NTC) and probes with target DNA (for OTR) were added to the alginate solution to make up the desired final concentrations of probes and target in 1% (w/v) alginate. For alginate liquid, 90 μl of this mixture was pipetted into wells of a 384-well microplate. For alginate hydrogel beads, 45 μl of this mixture was dropped into a bath of calcium chloride for 5 min. The beads were then removed, washed with distilled water then placed into individual wells of a 384-well plate, wherein each well contained 2 beads (90 μl total). Phosphate buffer (10 mM, pH 7.4) was used to buffer the gel.

## Fluorescence reading

Triplicate wells were prepared for each experiment. For each experimental condition, the fluorescence intensities of the non-templated control or NTC (probes without DNA) and the background coumarin or B (PNA-coumarin only) were also recorded. Unless otherwise indicated, fluorescence intensities were measured using a Fluorostar fluorescence plate reader (Labtech Omega, UK) ($\lambda_{exc}$= 485 nm, $\lambda_{em}$= 520 nm) at 4h from reaction initiation.

# S3. Optimisation of Alginate Hydrogel Conditions

**Figure S3. Optimising crosslinking conditions of 1% $Ca^{2+}$-alginate hydrogel beads for improved OTR efficiency, by varying (a) $CaCl_2$ crosslinking bath concentration and (b) crosslinking time (experiments conducted in triplicate).**

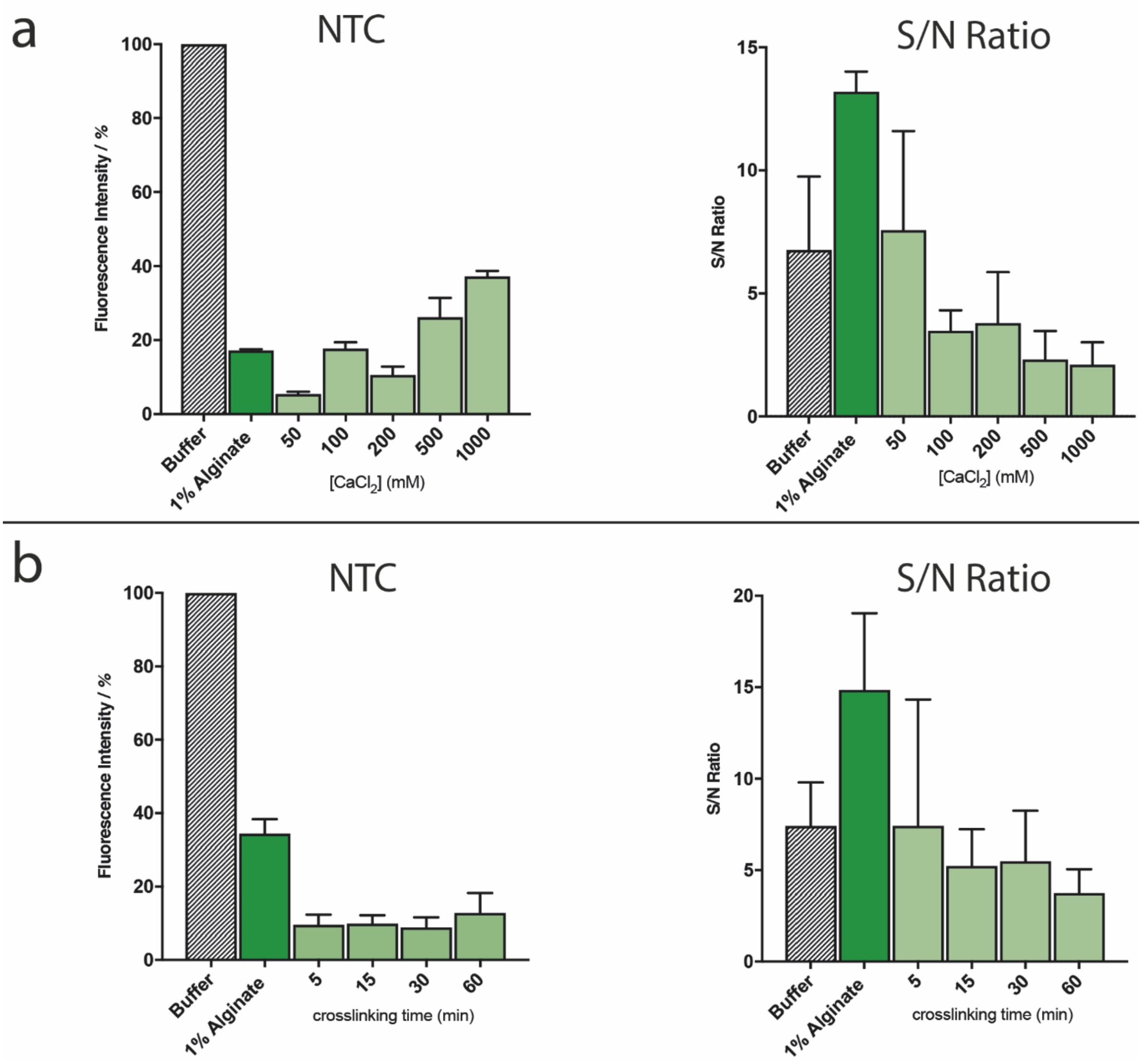


Oligonucleotide-templated reactions using a fixed stoichiometric concentration of PNA probes (5 µM each) and 1 µM of target NA were performed in alginate hydrogels, prepared using various crosslinking conditions. Beads of 1% (w/v) alginate, containing probes and target NA, were crosslinked in a bath of calcium chloride solution ($CaCl_2$) for a controlled period of time. To establish optimal conditions, the concentration of calcium was varied from 50 mM to 1 M. The lowest concentration (50 mM) of $CaCl_2$ resulted in the lowest NTC efficiency and accordingly highest S/N ratio; thus, it was chosen as the optimal condition for all future experiments. Similarly, the time allowed for alginate to crosslink within the bath of $CaCl_2$ was also varied from 5-60 min. The duration of 5-30 min crosslinking resulted in similarly low NTC efficiency and similar levels of S/N ratio; thus, the shortest duration of 5 min was chosen as optimal.

## S4. Kinetics of OTR in all media

**Figure S4. Investigating OTR kinetics in all media types (templated with 1 μM DNA) (experiment conducted in triplicate).**

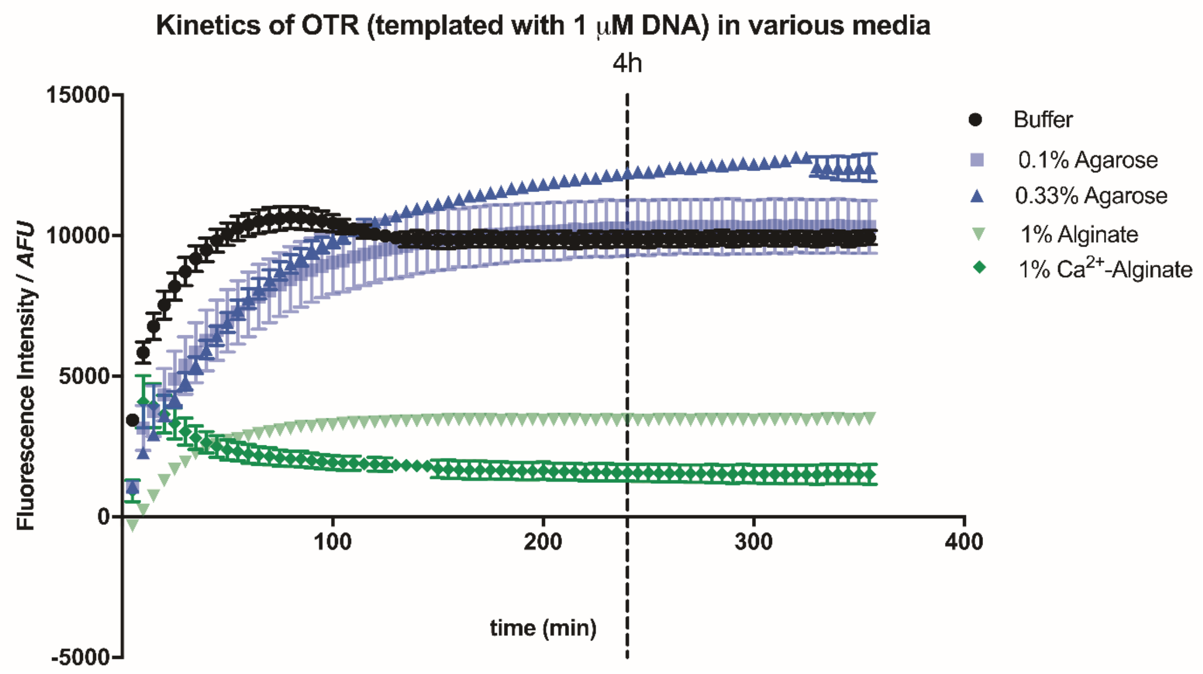


To investigate reaction kinetics, oligonucleotide-templated reactions using a fixed stoichiometric concentration of PNA probes (5 μM each) and 1 μM of target NA were performed in all media types (agarose, alginate and solution). The fluorescence intensity was monitored over a 6h period (See figure S4 above). By 4h from reaction initiation, equilibrium was reached in all the media investigated. Thus, for all further experiments, unless otherwise noted, the fluorescence intensity was measured at the single 4h time point from reaction initiation.

## S5. Optical Effects on measured fluorescence

Optical effects (such as scattering) within viscous and hydrogel media can reduce the fluorescence intensity measured. The following experiment was conducted to partially investigate this effect from each of the media. To measure these effects on the fluorescence of unquenched coumarin (i.e. product of OTR), a reaction was conducted in solution and allowed to reach equilibrium. After 5h, this reaction mixture was dispensed onto or added to the various media such that the final concentrations of probes and target DNA were 5 μM and 1 μM, respectively. The final media concentrations were 0.1% and 0.3% w/v for agarose, and 1% w/v for alginate (with or without added $CaCl_2$). To measure the optical effect on quenched coumarin (i.e. B), 5 μM of $PNA_1$ probe was similarly added to each medium. After incubation within each medium for 24h the fluorescence intensity was measured. Figure S5 shows the attenuation of OTR fluorescence intensity measured from the alginate and agarose media compared to solution. For each medium, the attenuation factors (i.e. ratio of intensity in solution to that in the agarose or alginate) were calculated and used as correction factors accounting for optical effects (e.g. in figure 1b).

**Figure S5. Assessing Optical effects on measured fluorescence in agarose or alginate compared to aqueous buffered solution (experiment conducted in triplicate).**

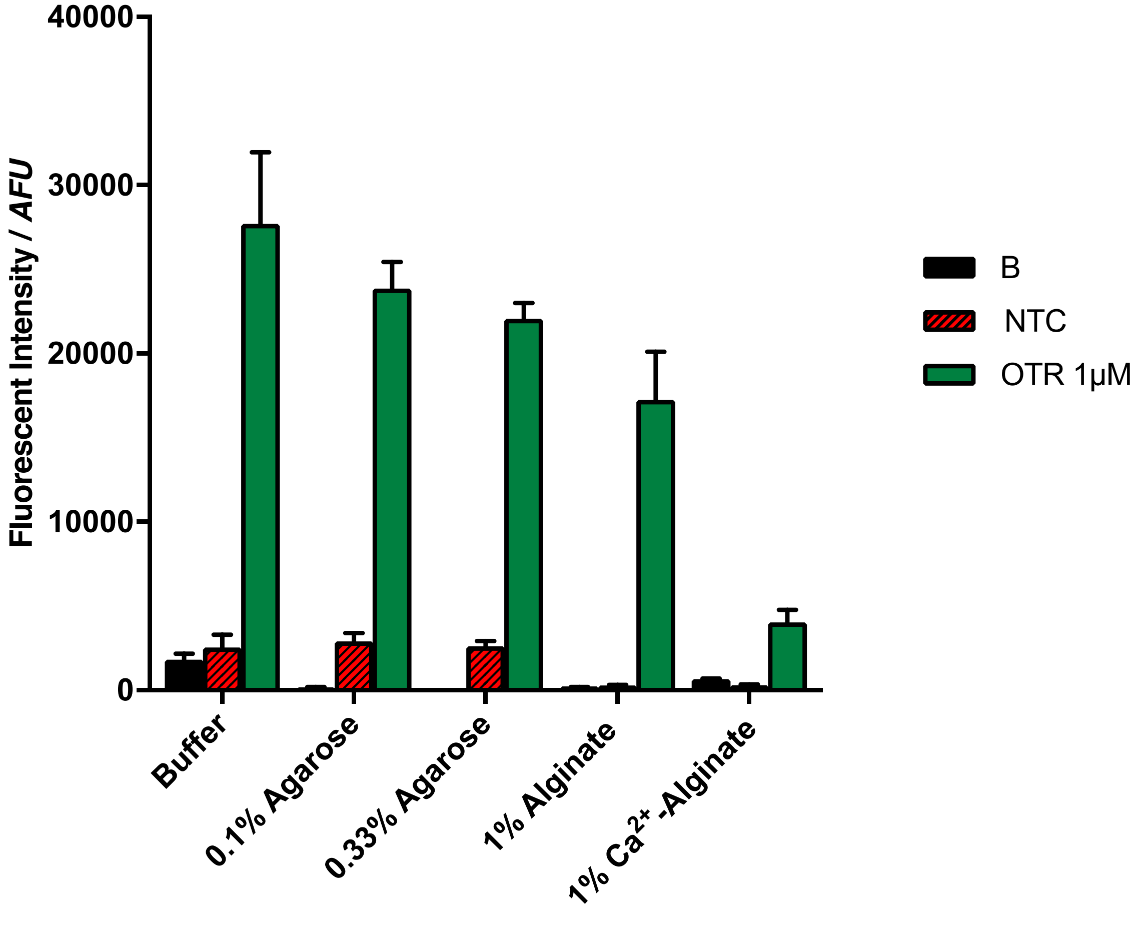

# S6. Estimating diffusion coefficient of PNA probes in Agarose

Low Melting Point (LMP) agarose gel (Sigma-Aldrich) was prepared by heating a suspension of LMP agarose in DNase- RNase-free water in a microwave for 40 s until fully dissolved. Approximately 1 mL was cast into a petri dish to produce a thin film of gel that was left to set for 20 min at RT. A maximum thickness of 1 mm was chosen so that the axial (z-dimension) diffusion could be neglected and diffusion could be simplified to two dimensions (planar diffusion). Fluorescein-labelled molecules of interest (1 μL) were then pipetted onto the film and allowed to diffuse for 5h. A Typhoon FLA9500 fluorescent scanner was used to image the plates at various time-points. Gain was set to PMT=300V, excitation wavelength to 473 nm and pixel size to 50 μm. The images obtained were analysed via Matlab software code. The resulting profile of fluorescence intensity $I(x,t)$ was fitted to a Gaussian curve following the equation below, and a diffusion coefficient (D) was extracted.

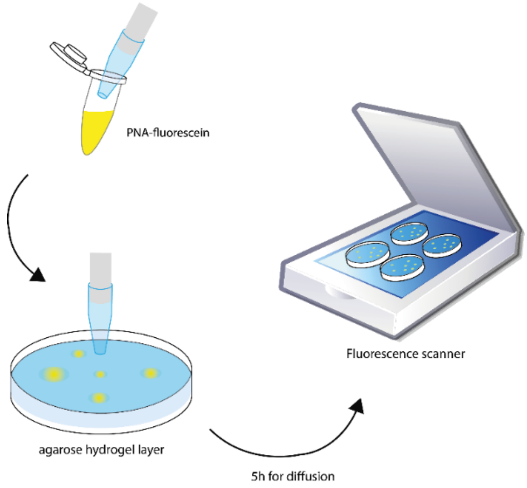


$$I(x,t) = \frac{F}{2\sqrt{\pi D t}} \cdot e^{-\left(\frac{x^2}{4Dt}\right)}$$

Where, $x$ = displacement of molecule from centroid (m)
$t$ = time (s)
$F$ = constant scaling factor
$D$ = estimated diffusion coefficient of molecule ($m^2$/s)

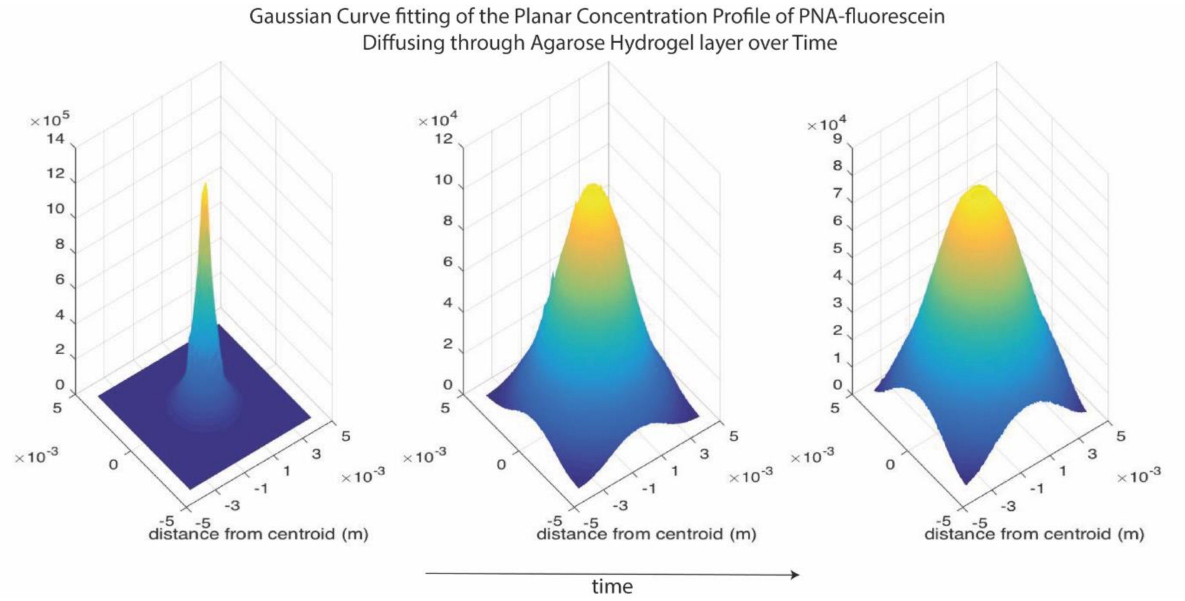


The molecule chosen to model our fluorescently-tagged PNA probe (MW = 2607 Da) is an 8 bp RNA molecule (MW = 2591 Da), whose dimensions and diffusion coefficient in solution can be easily derived from existing models and equations. Under low salt concentrations, the RNA adopts a hairpin conformation with hydrodynamic dimensions L = 2.6Å rise/bp x 4bp = 1.0 nm [2]. Considering previous observations on short DNA fragments, short RNA fragments (as herein) can also be modelled as a sphere [3]. Using a hydrodynamic radius of 1.0 nm, the diffusion coefficient in solution can be estimated from the Stokes-Einstein equation (at 20°C), to give $D_0=2x10^{-10}$ $m^2$/s. The diffusion coefficient in agarose gel was estimated using the

aforementioned experimental protocol and analysis to give $D_0$=1.2x$10^{-10}$ $m^2$/s, which is about 50% slower than that in solution.

## S7. Effect of Increasing probe concentration on LOD in 0.33% Agarose, 1% $Ca^{2+}$-Alginate and 1% Alginate

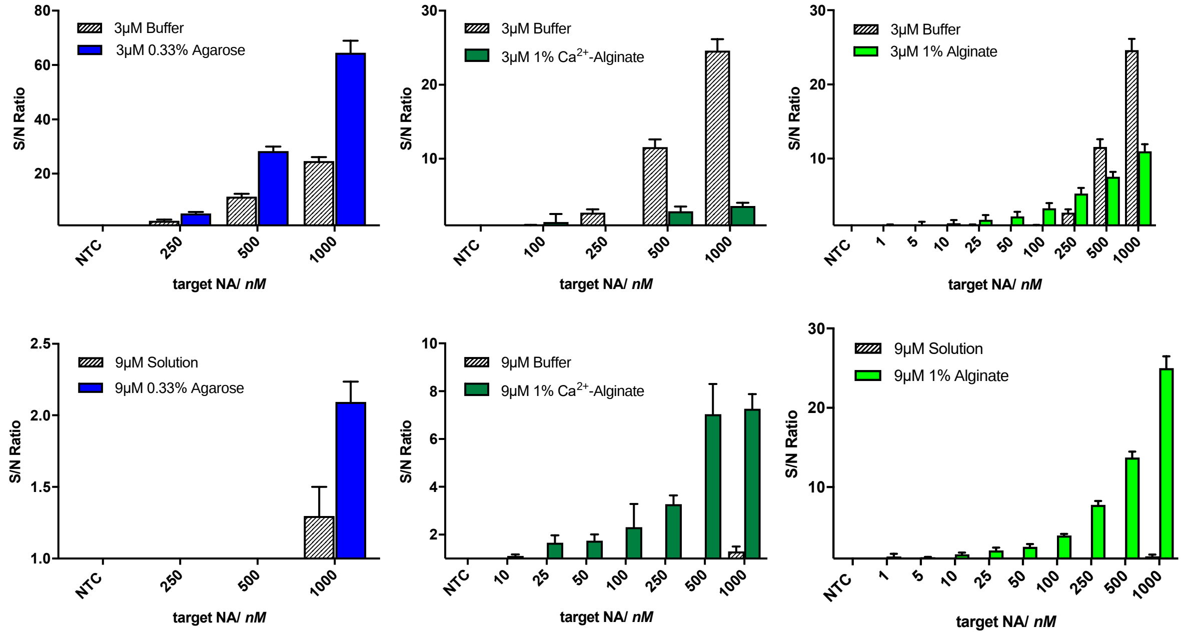


Sensing reactions were assessed using two different concentrations of PNA probes, at 3 μM and 9 μM each. For each condition, the DNA concentration was varied from 1 nM to 1 μM in order to establish the effect of probe concentration on sensitivity in the different media types. Using 3 μM probes, a linear correlation between fluorescence intensity and DNA concentration and a similar LOD (≈ 200 nM) were observed in solution and both hydrogel media (agarose 0.33% (w/v) and crosslinked $Ca^{2+}$-alginate bead). Increasing the probe concentration to 9 μM reduced the sensitivity in solution and agarose hydrogel setups. In alginate media however, a significant improvement in sensitivity was observed, where the LOD reduced to c.a. 50 nM in alginate bead and below 1 nM in non-crosslinked alginate. Figure S7 above shows the S/N ratio plots using both probe concentrations compared to a solution control (experiments conducted in triplicate).

## S8. References


[1] G. A. D. Metcalf, A. Shibakawa, H. Patel, A. Sita-Lumsden, A. Zivi, N. Rama, C. L. Bevan, S. Ladame, *Anal. Chem.* **2016**, *88*, 8091-8098.

[2] a) J. Lapham, J. P. Rife, P. B. Moore, D. M. Crothers, *J. Biomol. NMR* **1997**, *10*, 255-262; b) S. Arnott, D. W. L. Hukins, S. D. Dover, W. Fuller, A. R. Hodgson, *J. Mol. Biol.* **1973**, *81*, 107-122.

[3] W. Eimer, J. R. Williamson, S. G. Boxer, R. Pecora, *Biochemistry* **1990**, *29*, 799-811.
